# A assistência aos diabéticos em Portugal: o papel estruturante da Associação Protetora dos Diabéticos Pobres, 1926-1946

**DOI:** 10.1590/S0104-59702024000100007

**Published:** 2024-04-05

**Authors:** Ismael Cerqueira Vieira, João Rui Pita, Ana Leonor Pereira

**Affiliations:** i Investigador, Centro de Estudos Interdisciplinares do Século XX/Universidade de Coimbra. Coimbra – Portugal ivieira@uc.pt; ii Professor e investigador, Centro de Estudos Interdisciplinares do Século XX/Faculdade de Farmácia/Universidade de Coimbra. Coimbra – Portugal jrpita@ci.uc.pt; iii Professora e investigadora, Centro de Estudos Interdisciplinares do Século XX/Faculdade de Letras/Universidade de Coimbra.Coimbra – Portugal aleop@ci.uc.pt

**Keywords:** History, Diabetes, Assistance, Health, Portugal, História, Diabetes, Assistência, Saúde, Portugal

## Abstract

A assistência aos diabéticos em Portugal esteve umbilicalmente ligada à fundação da Associação Protetora dos Diabéticos Pobres em 1926, a qual teve um papel determinante na estruturação da diabetologia portuguesa. O objetivo do artigo é demonstrar de que forma a associação contribuiu para a estruturação de um modelo de assistência aos diabéticos em Portugal, escorada nos princípios da diabetologia moderna na primeira metade do século XX. Primeiro explicaremos como a instituição se organizou em termos funcionais e clínicos atendendo às políticas assistenciais do período do Estado Novo em Portugal. Posteriormente mostraremos como estava organizado o modelo de assistência, que incluía o tratamento e a educação dos diabéticos pobres.

## Abstract (en)

Assistance to diabetics in Portugal was quintessentially linked to the 1926 foundation of the Association to Protect Poor Diabetics (Associação Protetora dos Diabéticos Pobres) which was decisive in structuring diabetology in Portugal. This article shows how during the first half of the twentieth century, this association helped create a model of assistance for diabetics in this country based on the principles of modern diabetology. First, we examine its functional and clinical organization to comply with assistance policy during the New State period in Portugal. Next, we depict the organization of the assistance model, which included treatment and education of poor patients with diabetes.

A diabetes é atualmente uma das doenças que lideram as causas de morte em todo o mundo. Segundo a Organização Mundial de Saúde (WHO, 2016a), a prevalência e o número de pessoas com diabetes estão em crescimento não só nos países predominantemente ricos como nos países de rendimentos médios. A diabetes afeta na atualidade cerca de 422 milhões de pessoas no planeta, estimando-se que provoque anualmente cerca de 1,5 milhão de mortes (WHO, 2016b).

Até o século XX, a diabetes era uma doença relativamente rara e desconhecida fora da Europa e da América do Norte, para a qual havia falta de evidências epidemiológicas (Nwaneri, 2015). Do ponto de vista epidemiológico, 1/4 a 1/3 das mortes verificadas nos países ocidentais nas primeiras décadas do século XX deveram-se a doenças infetocontagiosas, decrescendo à medida que a difusão e utilização dos antibióticos tiveram lugar (Coste, 2014). O espaço deixado pela regressão das doenças infetocontagiosas foi ocupado por uma heterogeneidade de doenças de causas e mecanismos diversos, especialmente pelas doenças crónicas. A partir da década de 1920 as doenças cardíacas ocuparam um lugar destacado na mortalidade dos países ocidentais. O mesmo aumento verificou-se em doenças como o cancro, as cirroses alcoólicas do fígado, as broncopatias, as doenças metabólicas e da nutrição, das quais se salienta a *diabetes mellitus* (p.269-272).

O crescimento da *diabetes mellitus*, sobretudo diabetes tipo 2, verificou-se a partir da década de 1920 associada à obesidade e a anomalias lipídicas e favorecida por um modo de vida sedentário e uma alimentação demasiado rica em determinados nutrientes. A mortalidade, inicialmente estável, com taxas na ordem de dez por cem mil habitantes, atingiu, em meados do século XX, taxas de 25 por cem mil habitantes (Coste, 2014, p.272-273). Em casos mais específicos, como em Nova York, o aumento da mortalidade por diabetes foi mais precoce, sendo a mortalidade em 1923 de 22,9 por cem mil habitantes, 16 vezes mais elevada do que nos finais do século XIX (Hurley, 2010, p.52).

Desconhecem-se quais os números da morbilidade e da mortalidade por diabetes em Portugal até a década de 1930. Só na segunda metade do século XX assumiria algum relevo no panorama epidemiológico português. Até aí os poderes públicos insistiram mais no controlo sanitário das fronteiras, na organização e no aperfeiçoamento da luta contra as doenças infetocontagiosas e parasitárias e de doenças provocadas por carências alimentares, na higienização do meio ambiente (água de consumo, saneamento básico e habitação) e na profilaxia em geral (Ferreira, 1990, p.299; Costa, 1955).

Apesar de a diabetes passar despercebida aos poderes públicos nesse período, organizou-se em 1926 na cidade de Lisboa a Associação Protetora dos Diabéticos Pobres (APDP), uma instituição dedicada à assistência aos diabéticos pobres, de caráter particular e filantrópica. Além de ter organizado a assistência aos diabéticos em Portugal teve também nas suas origens funções como o estudo/investigação da doença, o diagnóstico e a educação dos doentes.

Quando a APDP foi criada não existia em Portugal nenhuma estrutura de assistência pública ou privada vocacionada para diagnosticar, acompanhar e tratar diabéticos. A própria assistência pública em geral era rudimentar e continuou a sê-lo durante décadas. Havia um afastamento do Estado das questões ligadas à saúde e assistência e uma afirmação da caridade particular em relação ao Estado. Somente na década de 1940 surgiu a primeira lei de bases da assistência social em Portugal, a lei n.1998, de 15 de maio de 1944.

Do ponto de vista médico, a diabetes estava mal estudada. Joaquim Diniz da Fonseca (1955), que foi subsecretário de Estado da Assistência (1940-1944) e presidente da APDP, dizia já na década de 1950 que havia um atraso sério no estudo da doença e que na Faculdade de Medicina de Lisboa eram apenas destinadas uma ou duas lições sobre o tema. Estudos recentes vieram mostrar que os trabalhos científicos e académicos produzidos em Portugal sobre diabetes eram escassos (Vieira, 2020; Vieira, Pita, Pereira, 2021). Por exemplo, do início do século XX até a descoberta da insulina em 1921 só foram produzidas sete teses de medicina cujo tema era a diabetes, no universo das três escolas médicas existentes em Portugal sediadas em Lisboa, Coimbra e Porto. Destas somente quatro teses eram de doutoramento. Os artigos científicos, publicados nas principais revistas médicas portuguesas, também foram escassos até a década de 1920.

A endocrinologia, como especialidade médica que veio a estudar, diagnosticar e tratar a *diabetes mellitus*, só foi organizada em 1949, com a fundação da Sociedade Portuguesa de Endocrinologia, e reconhecida em 1956 pela Ordem dos Médicos, isto é, a instituição que regula o exercício da medicina e concede carteiras profissionais aos médicos portugueses. Só a partir da criação dessa especialidade e da sua introdução nos *curricula* académicos foi consagrada maior atenção à doença ao nível universitário. Em Lisboa, no Hospital de Santa Maria, da Faculdade de Medicina da Universidade de Lisboa, o aparecimento da escola de diabetologia ocorreu somente em 1963, criada por Pedro Eurico Lisboa, que trabalhara sob a direção clínica de Ernesto Roma entre 1957 e 1963. Esse médico conseguiu mais tarde criar a Clínica de Diabetes e Nutrição nesse hospital, com uma estrutura multidisciplinar onde se especializaram médicos de medicina geral e familiar, endocrinologistas e internistas (Vieira, 2020, p.83). O estudo de Vieira (2020) revelou que, no caso da diabetologia, nos vários hospitais de Lisboa, a diferenciação foi tardia.

A APDP revelou ser um projeto pioneiro na conceção de um plano moderno de assistência e de tratamento aos diabéticos. O projeto foi liderado pelo médico Ernesto Roma, pioneiro da diabetologia no país. Nos inícios da década de 1920, Ernesto Roma, que estagiava na Harvard Medical School em Boston, assistiu ao que vulgarmente se apelida de milagre operado pela descoberta da insulina. Ernesto Roma aprendeu os conceitos e os métodos do trabalho social aplicados à medicina com o seu mentor, o professor Richard Cabot, praticando mais tarde esses conhecimentos em Portugal. Por outro lado, conheceu também o médico Elliott Joslin, o pioneiro no uso da insulina nos EUA. Do contato com Joslin, que criara a primeira clínica especializada no tratamento da diabetes nos EUA, aprendeu as bases para o seu tratamento. Foi o caso da insulinoterapia, da terapêutica alimentar específica, bem como da educação de doentes e da formação de profissionais de saúde envolvidos no tratamento e assistência.

Em Portugal são escassos os estudos sobre história da diabetes, incluindo o tratamento e assistência. Os estudos mais recentes sobre essa temática pertencem a Vieira, Pita e Pereira (2021), com publicações em torno da diabetologia social em Portugal intitulada “A diabetologia social em Portugal: génese, influências e concretizações (1926-1931)” e das “Influências norte-americanas na origem da diabetologia portuguesa na primeira metade do século XX” (Vieira, 2021), ambos focados no rastreamento das correntes de pensamento médico que influenciaram a diabetologia portuguesa. Ainda de Vieira (2020, 2018), as obras *A diabetologia em Portugal: contributos para a sua história* e *História da endocrinologia em Portugal no século XX*. Sobre a vida e obra do médico Ernesto Roma podemos destacar os trabalhos relativamente recentes de António Barros Veloso e Luís Gardete Correia (2017), *Ernesto Roma e a Associação dos Diabéticos Pobres,* e de Luís Gardete Correia e José Manuel Boavida (2006), *Fotobiografia de Ernesto Roma*.

Para este estudo foi identificado e analisado um *corpus* de documentos provenientes de várias instituições, fundamentais para perceber a evolução da primeira fase da assistência aos diabéticos em Portugal. Da APDP utilizamos algumas séries documentais como os Livros de Atas, em particular os volumes correspondentes a 1927-1929 e 1929-1938, os Estatutos da APDP, os sete números do volume 1 do *Boletim da APDP* (publicados sem regularidade entre 1931 e 1936) e publicações diversas da associação. Esse conjunto documental permite aceder à informação sobre a organização e a gestão da associação no seu quotidiano, as políticas seguidas, as opções estratégicas, e, de forma global, os eixos de atuação da instituição.

No Arquivo Histórico da Santa Casa da Misericórdia de Lisboa procedeu-se à consulta do *Livro de Atas do Conselho de Administração* para perceber as ligações e o apoio desta instituição à APDP no período estudado. Finalmente no Arquivo Histórico da Cruz Vermelha Portuguesa consultamos o *Boletim da Cruz Vermelha*, instituição que apoiou o trabalho da APDP no período da Segunda Guerra Mundial, por meio da sua rede internacional de contatos. Em termos metodológicos optamos pelo recurso a uma metodologia de tipo qualitativa baseada na pesquisa e análise documental e na inferência sistemática.

Com este artigo pretendemos preencher essa lacuna de estudos, examinando do ponto de vista histórico as principais fases do processo de estruturação de um modelo de assistência aos diabéticos em Portugal no período entre 1926 e 1946. Importa perceber como essa associação se organizou do ponto de vista funcional e clínico, num período em que a intervenção do Estado na assistência era reduzida, e como cumpriu o seu papel médico e social. Discutiremos seguidamente a lógica adotada pela associação no tratamento da diabetes na era da insulina e nas estratégias de educação de doentes e formação de profissionais de saúde, durante um período em que a diabetologia era uma área nova e em estruturação em Portugal como no mundo.

## As origens da Associação Protetora dos Diabéticos Pobres no contexto da assistência em Portugal

A idealização de uma associação que protegesse e socorresse os diabéticos pobres foi um projeto arrojado. Na década de 1920, quando foi criada a APDP, Portugal era um país com grande incidência de doenças infetocontagiosas que dominavam o panorama epidemiológico do país (Vieira, 2019). A tuberculose ocupava o primeiro lugar. Na década de 1920 estava-se longe de um quadro epidemiológico em que as doenças metabólicas e da nutrição tivessem destaque. Até 1931 a diabetes nem sequer aparecia mencionada na estatística obituária portuguesa de forma autónoma (Vieira, 2020, p.113), o que nos leva a concluir da sua diminuta importância em termos de mortalidade, possivelmente devido à sua raridade ou até de difícil ou impreciso diagnóstico e também por se associarem à diabetes outras patologias mortais. Também é certo que as estatísticas consultadas incidem sobretudo sobre a mortalidade. Esse estudo está muito bem retratado no primeiro terço do século XX na tese de doutoramento *Portugal sanitário: subsídios para o seu estudo* (Correia, 1938), em que a tuberculose surge como primeira causa de morte.

O projeto da APDP partiu da iniciativa de Ernesto Roma, médico do Hospital Escolar de Santa Marta, em Lisboa, que em 1922 se estabeleceu em Boston, nos EUA, a fim de se especializar em ética com o professor Richard Cabot. Richard Cabot foi um médico, filósofo e diretor do departamento de Ética da Universidade de Harvard. Foi um importante cultor do trabalho social nos EUA, tendo organizado em 1905 o primeiro departamento de serviço social num hospital norte-americano. O Departamento de Ética Social, presidido por Cabot, passou a ensinar ética aplicada aos estudantes de medicina de Harvard, integrando nos seus *curricula* o trabalho médico-social no Massachussets General Hospital (Richmond, 1917, p.36; Garchar, Kaag, 2013, p.556).

No mesmo período, em Boston, organizou-se a formação de enfermeiras especialistas em diabetes no New England Deaconess Hospital School of Nursing, onde lecionava Elliott Joslin. A partir de 1922, foi posto em prática um programa rigoroso de formação de enfermeiras capazes de intervirem junto dos doentes e suas famílias e providenciarem uma educação diabetológica tão completa quanto possível (Barnett, 1998, p.43). Essa educação diabetológica compreendia o ensino da higiene em geral, o ensino da culinária aplicada ao estado de saúde dos diabéticos, a técnica de autoinjecção de insulina, a análise das urinas pelos próprios doentes etc.

Ernesto Roma colheu importantes ensinamentos na área do trabalho social aplicado à medicina, que mais tarde implementou na APDP. Aliás, a origem da APDP como associação destinada ao tratamento de doentes pobres testemunha a vocação social da instituição. Mas houve também um trabalho importante realizado em nível do empoderamento dos diabéticos com o ensino da higiene, da dietética e da insulinoterapia e ainda com a visitação, o acompanhamento e o aconselhamento domiciliário aos doentes por parte de visitadoras.

Previamente à fundação da APDP, Ernesto Roma já fazia uso dos conhecimentos adquiridos nos EUA. Ao regressar a Portugal nos finais de 1923 terá sido o primeiro médico português a tratar doentes com insulina, mas rapidamente percebeu que se sabia pouco acerca da diabetes. Verificou que era necessário oferecer aos médicos informação atualizada sobre a doença e sobre o seu tratamento (Veloso, Correia, 2017).

A consciência de que a diabetes era uma doença crónica e sem cura exigia um tratamento colaborativo, em que o doente era peça-chave. Era fundamental que o doente soubesse comer adequadamente, autoinjetar a insulina, controlar a glicosúria, ser autónomo e reintegrar-se na sociedade. Essa visão implicava uma educação alargada dos diabéticos com o ensino da higiene, da dietética, da insulinoterapia e a aprendizagem e domínio de toda uma tecnologia da diabetes (Gardner, 2019).

Ernesto Roma pôs em prática essa educação dos diabéticos na clínica privada, junto de uma clientela burguesa e abastada. Contudo, estava consciente de que existia um número crescente de diabéticos que não tinham qualquer tipo de apoio e assistência médica. Essa falta de apoio era motivada por ausência de uma organização de assistência específica e por falta de conhecimento dos médicos, o que foi ultrapassado com a criação da APDP. A APDP revelou-se um projeto pioneiro em nível mundial (IDF, 1978, p.7), já que associações do mesmo tipo só se constituíram cerca de uma década mais tarde. São disso exemplo as associações de diabéticos da Tchecoslováquia e da Inglaterra fundadas em 1936, seguindo-se o aparecimento de associações congéneres em França em 1938, nos EUA em 1940, na Bélgica e na Suécia em 1942, na Austrália, Países Baixos e Itália em 1945 e na Noruega em 1948 (Costa, 1955, p.154).

Em 1926, aquando da fundação da APDP em Lisboa, vivia-se em Portugal um período de grande instabilidade política e económica. Essa instabilidade era devida ao fracasso do projeto revolucionário e modernizador que a revolução republicana de 1910 tentou implementar em Portugal (Farinha, 2011). No âmbito das estruturas de saúde e assistência, Portugal revelava um certo atraso em face de outros países europeus.

Somente nos inícios do século XX teve lugar a primeira grande reforma sanitária, liderada pelo sanitarista Ricardo Jorge, com a reorganização da Direção-geral de Saúde e Beneficência Pública em 1903 (Ferreira, 1990, p.337-338). Mas poucas foram as concretizações da reforma arquitetada por Ricardo Jorge, quer por falta de interesse e dinheiro, quer pela ausência de políticas sociais adequadas. Como defende Abreu (2018, p.17),

a maioria da população oscilava entre a pobreza e a miséria, com muitas doenças ainda endémicas em Portugal, quando já recuavam noutros países da Europa. … a pobreza constituía o principal problema de saúde pública, e não era com paliativos que se fazia uma revolução.

No âmbito da saúde existia um atraso secular que se devia ao subfinanciamento estatal e aos apelos dos poderes políticos à caridade privada, que nem a Revolução Liberal nos inícios do século XIX nem a implantação da República em 1910 conseguiram resolver. As políticas sociais em matéria de educação, saúde, emprego, habitação e segurança social modernas tiveram de esperar até 1974 para começar a ver a luz do dia, com a constituição de um verdadeiro Estado-providência. Como refere Pereira (1999, p.45-61), antes da ditadura do Estado Novo português a proteção social repousava em movimentos mutualistas voluntários e organizações afins, no paternalismo patronal de raiz católica ou filantrópica e no seguro obrigatório instituído em 1919. Por outro lado, existia uma retórica antissocial da ditadura, ou melhor, “antissocialista”, o que empurrou a dimensão social do sistema corporativo para a dependência da ação das misericórdias e mutualidades no domínio da assistência social (Garrido, 2018, p.199-200).

Assim, o período histórico em que ocorreu foi um período dominado por convulsões políticas, sucessivos défices financeiros e mau desempenho económico. Várias leis e regulamentos no campo da saúde e assistência não passaram do papel. Até 1946, o modelo de assistência pública tinha uma intervenção estatal mínima e supletiva em relação às instituições privadas, onde se incluíam as misericórdias, as associações mutualistas e as autarquias (Campos, 2013; Alves, Carneiro, 2013).

Na década de 1920, quando surgiu a APDP, a medida mais assinalável no campo da assistência foi a reorganização da assistência pública por meio do decreto n.11.267, de 25 de novembro de 1925. Legalmente a Direção-geral de Assistência ficou dependente do Ministério do Interior. A Provedoria Central da Assistência de Lisboa ficou a controlar a Casa Pia de Lisboa, a Misericórdia de Lisboa, os hospitais da Universidade de Coimbra e mais dois hospitais nas Caldas da Rainha. No ano seguinte teve lugar a reorganização geral dos serviços de saúde pública pelo decreto n.12.477, de 12 de outubro de 1926. Esse decreto, de cunho marcadamente sanitarista, tinha como objetivos a elaboração da estatística sanitária, a sanidade marítima e terrestre, a prevenção das doenças de foro infecioso, a higiene do meio laboral e escolar, bem como a inspeção alimentar. Como seria de esperar, não existiu nenhum tipo de preocupação com a diabetes, que tinha uma baixa incidência na população, a julgar pelas características epidemiológicas daquela época.

Como era expectável, o problema do tratamento da diabetes era duplo: por um lado, a falta de informação e conhecimentos médicos sobre a doença e seu tratamento; por outro, a falta de instituições vocacionadas para assistência a esse tipo de doentes. A APDP veio responder a esses dois problemas criando uma associação cujos objetivos principais passavam por fornecer aos diabéticos a insulina e outros medicamentos, divulgar preceitos científicos modernos sobre o tratamento e ao mesmo tempo proteger e socorrer os diabéticos reconhecidamente pobres (APDP, 1927).

Na década de 1930, a legislação corporativa do Estado Novo em Portugal^1^ estabeleceu a Previdência Social e a Assistência Social como sendo duas vias institucionalmente distintas. Para aqueles que exerciam uma atividade laboral, como os trabalhadores da indústria, comércio ou serviços, existia um sistema de seguro social obrigatório designado de Previdência Social; para aqueles que não tinham capacidade contributiva, a Assistência Social prefigurava-se como a única opção (Alves, Carneiro, 2013, p.335). Como já referimos, o decreto n.11.267, de 25 de novembro de 1925, incumbira a Direção-geral de Assistência de tutelar as instituições de assistência públicas, que se resumiam a algumas instituições sediadas em Lisboa, Coimbra e Caldas da Rainha. O resto do país só alcançava a assistência por meio das autarquias locais e de entidades particulares como as misericórdias e as associações mutualistas (p.340).

O projeto de uma associação destinada a socorrer diabéticos pobres era convergente com as políticas de saúde das décadas de 1920-1930 e subsequentes, em particular com as políticas de saúde do Estado Novo a partir da Constituição da República, de 1933. Na década de 1930, os países europeus passaram a considerar a saúde da sua população um dever do Estado. Na mesma época o governo português baseava apenas a sua ação na promoção e fiscalização das instituições privadas de assistência (Almeida, 2017, p.105), regulamentando no sentido de serem as câmaras municipais, ou seja, os poderes locais, a custear o tratamento dos doentes pobres (decreto-lei n.23.348, de 13 de dezembro de 1933).

O Estado português considerava os doentes pobres não só os indigentes como ainda todos os indivíduos impossibilitados de trabalhar, sem recursos para sobreviver e sem família que os pudesse ajudar ou prestar auxílio. Mas eram também considerados doentes pobres aqueles que viviam exclusivamente do seu trabalho e que dele auferissem apenas o indispensável para a sua manutenção e das pessoas a seu cargo (decreto-lei n. 23.348, de 13 de dezembro de 1933). Quer isto dizer que, para o Estado Novo, uma significativa parte da população se enquadrava nessa categoria de pobre, o que implicava o recurso a determinadas instituições de beneficência particular para atenderem ao tratamento da sua doença.

A ação da APDP em Lisboa e nas cidades onde tinha filiais enquadrava-se perfeitamente nesse esquema de assistência social definido e promovido pelo Estado Novo. Por isso existia uma função de beneficência subjacente à atividade da APDP, bem como existia uma lógica de profilaxia da doença e assistência material aos doentes, com o fornecimento de insulina, medicamentos, seringas, agulhas, alimentos etc.

A análise dos *Livros de Atas* da APDP (1927-1929) deixa perceber que nos inícios da sua atividade vivia essencialmente de donativos, de ofertas de particulares ou de instituições e das quotas dos sócios. Em 1927, no primeiro ano de trabalho efetivo da APDP, verificaram-se as primeiras ajudas de particulares: a companhia farmacêutica Eli Lilly e o Instituto Pasteur de Lisboa ofereceram cerca de 4.500 unidades de insulina cada; a empresa Coll Taylor ofereceu uma redução no preço da insulina; a empresa portuguesa Cutelaria Policarpo em Lisboa fez preços especiais na aquisição de agulhas e seringas; e a 31 de outubro desse ano a Santa Casa da Misericórdia de Lisboa decidiu estabelecer um subsídio mensal de cinco mil unidades de insulina à APDP, durando essa parceria várias décadas (Ata…, 31 out. 1927, p.574). Mas não eram só as pessoas a título individual que pediam auxílio da APDP. Também os hospitais públicos encaminhavam os diabéticos pobres para a associação, como aconteceu com o Hospital de Santa Marta em Lisboa, que, em 1927, encaminhou o primeiro diabético para a APDP para receber tratamento com insulina (APDP, 1927-1929, p.18).

Em 1940, foi criado o Subsecretariado de Estado da Assistência Social dentro da estrutura do Ministério do Interior. Tratou-se de um departamento destinado a coordenar todas as atividades de assistência e saúde dispersas pelos vários ministérios (Almeida, 2017, p.125). Foi seu primeiro subsecretário Joaquim Diniz da Fonseca, que mais tarde integrou a APDP na qualidade de presidente da Direção, de 1952 a 1958 (Vieira, 2020, p.133).

Quatro anos mais tarde, a lei n.1998, de 15 de maio, estabeleceu as bases reguladoras dos serviços de assistência social. No seu essencial, a lei n.1998 (Base III) manteve um caráter supletivo em relação aos particulares, afirmando que cabia ao Estado central ou às autarquias suscitar, promover ou sustentar obras de assistência apenas na falta ou insuficiência das iniciativas particulares.

Finalmente, em 1945, o governo português levou a cabo uma reorganização dos serviços de assistência social (decreto-lei n.35.108, de 7 de novembro), que pretendeu implementar novas ideias e alargar funções e objetivos, dos quais se destacam a assistência preventiva, destinada a combater vários flagelos sociais, ou a assistência construtiva, destinada a melhorar a qualidade de vida das populações. Apesar desse alargamento de funções dos serviços de assistência social, podemos afirmar que a diabetes foi deixada de parte como problema social. Nesse decreto-lei só é feita menção às doenças infetocontagiosas, oncológicas e psiquiátricas, ainda que no ano que antecedeu a sua promulgação as doenças da nutrição, e em particular a diabetes, tivessem sido debatidas na Assembleia Nacional, tendo-se enfatizado o número crescente de óbitos ocorridos devido à diabetes nos anos anteriores (Portugal, 1944, p.102).

Cerca de 1945, num momento histórico no qual vários países europeus se preparavam para montar o Estado-providência, Portugal manteve um sistema de assistência social mínimo e excessivamente dependente da iniciativa particular. O Estado português atuava meramente com o intuito de suprir as necessidades e os vazios deixados pela iniciativa privada.

Nesse contexto, a luta contra a diabetes e o tratamento dessa doença não tiveram lugar no programa da Direção-geral de Assistência. Foi em grande medida a ação desenvolvida pela APDP e pelas suas filiais espalhadas por alguns pontos do país que prestou auxílio e assistência aos doentes diabéticos portugueses.

## Estrutura e organização interna da associação

A ideia de criar uma associação de luta contra a diabetes em Portugal partiu do médico Ernesto Roma. A sua passagem pela Universidade de Harvard no momento em que a insulina era a grande descoberta mundial no campo médico e os contatos que fez com médicos norte-americanos na área do serviço social e diabetologia terão marcado decisivamente esse médico português. Entre a chegada a Portugal e a criação da APDP existiu um período de cerca de três anos em que não existem registos de qualquer iniciativa de caráter assistencial no campo da diabetes. Veloso e Correia (2017) referem que durante esse período Ernesto Roma trabalhou quer no Hospital de Santa Marta em Lisboa quer na clínica privada, tratando doentes segundo os métodos aprendidos nos EUA. Devemos referir que não encontramos evidências desses fatos pela inacessibilidade à documentação.^2^

A ideia de criar uma associação de assistência aos diabéticos pobres terá sido concebida presumivelmente entre 1923 e 1926. A ideia de filantropia acompanhou Ernesto Roma durante toda a vida. Em criança conviveu de perto com o exemplo do avô materno, o doutor Policarpo Galeão, que era médico militar no Regimento de Infantaria 3 em Viana do Castelo, cidade do norte de Portugal. O avô de Ernesto Roma era conhecido como o “pai dos pobres” em Viana do Castelo porque prestava serviços médicos gratuitos e dava esmolas às famílias carenciadas dos bairros piscatórios. Aquando da morte do avô, em 1905, Ernesto Roma recebeu um bilhete do pai, o general Bento Roma, acompanhado de um recorte de jornal noticiando a morte do avô. No bilhete, que guardou toda a vida e se encontra atualmente à guarda do Arquivo Municipal de Viana do Castelo, pode ler-se: “Tua avó envia-te este bocado de jornal, para que tu e o João o leiam com atenção; e o guardem; a fim de seguirem sempre o exemplo do seu Avô. B. Roma” (AMVC, 1905). Assim, colocamos como hipótese poder ter sido a ideia de filantropia, acompanhada do desejo de pôr em prática os conhecimentos recentes sobre o tratamento da diabetes, os principais motivos para a fundação da APDP. Em entrevista ao periódico *Notícias Médicas* (Entrevista..., 1976, p.8-9), Ernesto Roma declarou que, em maio de 1926, no final de uma palestra com os seus doentes da clínica privada, apresentou a ideia de criar uma associação vocacionada para a educação dos diabéticos e que prestasse auxílio aos doentes pobres que não dispunham de meios para ter acesso a uma assistência especializada. A ideia colheu adeptos e, após alguns meses de preparativos, realizou-se a 28 de agosto de 1926 a primeira assembleia-geral constituinte da APDP, na presença de 21 sócios fundadores (Vieira, 2020, p.131-132).

A APDP definiu-se desde o início como uma instituição humanitária e beneficente destinada a socorrer e proteger todos os doentes diabéticos reconhecidamente pobres, independentemente de sexo, idade e condição. Essa ajuda concretizou-se por meio de um pacote de medidas que incluía o fornecimento de insulina e outros medicamentos, instrumentos como balanças e géneros alimentares e divulgação de preceitos científicos modernos acerca do tratamento da doença. A sua ação previa ainda a criação de bolsas para apoiar estudos e experiências científicas no ramo da medicina.

Nos anos seguintes, a APDP alargou seu raio de ação geográfica, passando a intervir não só nas cidades do distrito de Lisboa como em vários pontos do território português por meio de filiais, delegações ou postos e ainda, virtualmente, nas ilhas adjacentes dos Açores e da Madeira, bem como nas colónias ultramarinas.

A APDP alargou igualmente a sua ação funcional e assistencial. No âmbito funcional ficou responsável por organizar cursos da especialidade ou dietéticos destinados a médicos e enfermeiras, uma biblioteca e um museu. Outra finalidade era a de manter relação com instituições estrangeiras congéneres e com entidades nacionais ou estrangeiras produtoras de drogas ou produtos alimentares para diabéticos para facilitar a obtenção desses produtos nas melhores condições.

Já vimos que em nível assistencial estava em consonância com a nova Constituição de 1933. Passou formalmente a proteger doentes indigentes, pobres ou necessitados e a proceder à hospitalização de doentes diabéticos pobres em hospital privativo na sua sede, e ainda de doentes não pobres mediante o pagamento dos cuidados médicos e internamento.

Verificamos que ao longo dos anos a instituição foi acompanhando as transformações no campo da assistência, adaptando-se à nova realidade governativa do Estado Novo. Além de tratar e educar os diabéticos pobres, os estatutos da APDP permitiam alargar os seus objetivos e o seu raio de ação. Desse modo passou também a considerar sua finalidade a formação de médicos e profissionais de enfermagem no campo da diabetologia e da dietética, em face da inexistência de formação específica no país. Alargava ainda a sua ação a outros territórios quer no continente quer nos arquipélagos portugueses da Madeira e dos Açores ou ainda nas colónias em África e na Ásia.

Em relação à organização interna, a associação iniciou atividade sob um regime jurídico diferente do que adotaria a partir de 1933. Os *Estatutos* de 1927 (APDP, 1927) tinham como órgãos administrativos uma assembleia-geral e uma comissão executiva.

A Assembleia-geral era composta por todos os sócios contribuintes maiores de idade. Pagavam uma quota superior a cinco escudos (moeda portuguesa da época). Era um órgão regulamentador e deliberativo. A Comissão Executiva tinha por função gerir a associação, admitir novos sócios, elaborar regulamentos internos, escolher o diretor técnico, contratar e despedir empregados, entre outras funções.

Em 1928, houve uma revisão dos *Estatutos* (APDP, 1928), que pouco mexeu nas questões do foro administrativo. Foi apenas criada a função de consultor jurídico, que tinha como principais incumbências orientar os serviços da associação dentro da lei e manter relações com as várias entidades oficiais, além de emitir pareceres de caráter administrativo e executivo.

O início da ditadura em Portugal levou à entrada em vigor de uma nova Constituição em 1933. No mesmo ano, a APDP alterou os seus *Estatutos* (APDP, 1933) para estar conforme às novas disposições legais. A gestão administrativa passou a repousar em quatro órgãos: a Comissão Central, o Conselho de Administração, a Direção e a Junta Consultiva. No topo estava a Comissão Central, composta por quarenta sócios escolhidos e nomeados pela Direção-geral da Assistência, sendo na prática uma ramificação que o governo da ditadura mantinha na APDP. Competia-lhe enviar à Direção-geral de Assistência a lista dos sócios da APDP, exercer a ação disciplinar, eleger sócios para lugares vacantes, apreciar e votar o relatório anual, autorizar a criação de novos serviços, propor alterações aos estatutos e autorizar a criação de filiais.

O Conselho Administrativo era uma extensão da Comissão Central composta por sete membros, com funções complementares: elaboração do relatório anual de contas, aprovação das contas das novas filiais, organização de orçamentos e promoção de inspeções a filiais, delegações e postos. A Direção manteve, na generalidade, as mesmas funções. Foi criada uma Junta Consultiva para dar pareceres ao diretor clínico e presidente da associação, propor ao ministro do Interior o diretor clínico e seu substituto e ainda votar e propor ao mesmo ministro a exclusão de qualquer membro da instituição.

A estrutura diretiva da associação voltaria a ser alvo de remodelação estatutária em 1939, compondo-se de uma Assembleia de Sócios, que na prática era uma Assembleia-geral, uma Direção e uma Comissão de Contas (APDP, 1939).

Apesar dessas alterações na organização e estrutura da APDP, entre o início da sua atividade e 1946, houve uma figura sempre presente e com as mesmas atribuições: o diretor clínico, mais concretamente o doutor Ernesto Roma. A figura do diretor clínico representava o cerne da atividade da associação, por várias razões: primeiro porque era inicialmente o único médico a exercer funções, e segundo porque era dos raros médicos especializados no tratamento da diabetes em Portugal.

O diretor clínico, também designado de diretor técnico, era figura de relevo dentro da associação, por ser o fundador da APDP e o mentor desse projeto, que abriu novos horizontes tanto médicos como assistenciais no plano do tratamento da *diabetes mellitus*. Há que contar também com o fato de estatutariamente o diretor clínico ter de ser um especialista em diabetes, o que marcava a diferença pelos conhecimentos que exigia. Lembre-se que a diabetologia nunca atingiu o estatuto de especialidade em Portugal.

Dentro da estrutura associativa, o diretor clínico dirigia sob o ponto de vista médico todos os serviços, devendo igualmente elaborar estudos sob o ponto de vista técnico e dar parecer à Comissão Executiva sobre os assuntos de caráter clínico. Do ponto de vista assistencial era ele que dava parecer sobre os pedidos de auxílio feito pelos diabéticos, pelo que decidia se o diabético cumpria ou não os requisitos para ser auxiliado.

A partir de 1933, o diretor clínico passou a ter algumas outras funções: a escolha do pessoal clínico, farmacêutico e de enfermagem, a direção do boletim e de outras publicações clínicas ou de propaganda, indicar o seu sucessor no cargo e elaborar regulamentos clínicos. Podia ainda, em casos justificados, substituir o presidente da APDP. Em 1939, assumiu também a direção do laboratório, a hospitalização, o refeitório dietético, a farmácia, a biblioteca e o museu.

## A estruturação do modelo de assistência na era da insulina

À data da fundação da APDP, a diabetologia como área médica inexistia em Portugal (Vieira, Pita, Pereira, 2021). De acordo com Weisz (2006, p.15), o processo de especialização em medicina desenvolveu-se não só com a investigação e ensino, mas também com o interesse público num determinado problema de saúde que levava à criação de instituições votadas ao seu tratamento e ao desenvolvimento de especialidades. Em Portugal, o interesse existente parece ter partido essencialmente de particulares, sobretudo os afetados pela doença, mas nunca gerou um interesse público suficiente para elevar a diabetologia à categoria de especialidade. Do ponto de vista científico e clínico, a diabetologia veio a ser alvo de integração pelas especialidades de medicina interna e de endocrinologia-nutrição, mas num período posterior àquele que nos propomos estudar aqui (Vieira, 2018).

Alguns autores como Botelho (1988) e Ruas (2017) são unânimes em mostrar que foi em Lisboa que a diabetologia surgiu e se tornou referência para o país, primeiro pelo trabalho desenvolvido pela APDP, desde 1926, e depois pela Escola de Diabetologia do Hospital de Santa Maria/Faculdade de Medicina da Universidade de Lisboa a partir de 1963, liderada por Pedro Eurico Lisboa. A atenção dada à diabetologia noutros espaços de referência surgiu tardiamente em comparação com Lisboa.

Em Coimbra foi dada uma particular atenção à diabetologia por Manuel Bruno da Costa, professor da Faculdade de Medicina da Universidade de Coimbra. Bruno da Costa cedo se interessou pela endocrinologia, sendo mesmo o primeiro a produzir insulina em Portugal no Laboratório de Química Biológica da referida universidade (Costa, 1938). Do ponto de vista assistencial foi inicialmente o responsável clínico da filial da APDP em Coimbra, dando consultas aos diabéticos pobres. De acordo com Carrilho, Ruas, Carvalheiro (2014, p.15), por razões de burocracia universitária, Bruno da Costa passou para o serviço de doenças infetocontagiosas da Faculdade de Medicina, perdendo a ligação à causa da diabetes. Só na década de 1960, com o médico Almeida Ruas, a endocrinologia entrou decisivamente no ensino universitário, por meio de cursos de endocrinologia e doenças metabólicas associados à cadeira de patologia médica. Mas somente em 1973 foi criada uma consulta externa de endocrinologia nos Hospitais da Universidade de Coimbra, onde a diabetes também era tratada (p.18-20).

No Porto, a primeira consulta de endocrinologia foi criada em 1953, por iniciativa do médico Ignácio Salcedo, no Hospital Geral de Santo António (Silva, 2003), que estava à data associado à Faculdade de Medicina da Universidade do Porto. Era na consulta de endocrinologia que se fazia o tratamento da *diabetes mellitus* em contexto hospitalar, se bem que a primeira iniciativa para o tratamento dos diabéticos pobres na cidade do Porto partiu da APDP, por intermédio da sua filial criada em 1928.

Os fatos descritos permitem-nos constatar que, na primeira metade do século XX, a grande impulsionadora da diabetologia em Portugal, na vertente assistencial e clínica, foi a APDP e as suas filiais. Não encontramos na história dos hospitais de referência em Portugal nem nas faculdades de medicina uma linha de trabalho ligado à diabetologia na primeira metade do século XX.

No contexto internacional, desde o século XIX tinham sido dados passos importantes no diagnóstico da doença, especialmente com a invenção e aperfeiçoamento dos testes de deteção da glicose na urina e no sangue por investigadores como Karl August Trommer, Herrmann von Fehling e Karl Gotthelf Lehmann (Tattersall, 2009; Schadewaldt, 1989).

A capacidade de determinar a quantidade de açúcar na urina, e depois no sangue, fez evoluir a terapêutica dietética, que era a única terapêutica verdadeiramente lógica até a descoberta da insulina. Como forma de fazer baixar a glicosúria eram prescritas dietas restritas em hidratos de carbono desde o século XVIII. Essas dietas foram aperfeiçoadas nos inícios do século XX por vários investigadores norte-americanos como Frederick Allen (1913) e Allen, Edgar Stillman e Reginald Fitz (1919), e depois usadas por grandes nomes da diabetologia como Elliott Joslin.

O avanço decisivo no tratamento da diabetes deu-se com a descoberta da insulina em 1921-1922 (Bliss, 2017) por Frederick Banting e Charles Best. A comercialização em larga escala dessa hormona permitiu estender o tratamento pela insulina a vários pontos do globo e salvar a vida a milhões de diabéticos.

Tinham apenas decorrido quatro anos desde a primeira utilização da insulina em humanos, quando foi criada a primeira associação portuguesa dedicada a tratamento, educação e assistência aos diabéticos pobres em Portugal. Mas o projeto inicial da APDP tinha um objetivo simples: o de fornecer aos diabéticos pobres a insulina que precisavam para o seu tratamento diário. A instituição foi criada e organizada de acordo com a visão do seu fundador, o doutor Ernesto Roma (Veloso, Correia, 2017).

No período entre 1926 e 1946 a diabetologia em Portugal estruturou-se essencialmente em torno do projeto da APDP. Inicialmente, a ação da APDP estava limitada a Lisboa, mas pouco depois passou a dar assistência a outras cidades portuguesas. A importância da ação assistencial da APDP era tão grande que alguns hospitais da capital, como o Hospital Escolar de Santa Marta e o Hospital de São José, enviavam os doentes diabéticos para receber tratamento ambulatório na APDP (APDP, 1927-1929, p.18).

O tratamento da diabetes exigia conhecimentos médicos específicos e um programa de ensino aos doentes que poucos conheciam. O tratamento da diabetes compreendia administrar insulina nas doses certas e a existência de um plano alimentar e higiénico rigoroso. A descoberta e o emprego da insulina revelaram-se, a médio prazo, insuficientes, gerando muitas complicações (Tattersall, 2009, p.82), como a retinopatia diabética, a nefropatia diabética, gangrenas, outros problemas de foro vascular, impotência sexual etc.

Na década de 1920, quando o mundo pouco sabia sobre o tratamento e assistência aos diabéticos, a APDP iniciou o seu trabalho médico e social com conhecimentos vanguardistas para a época. Numa época em que os médicos portugueses seguiam atentamente os progressos da medicina francesa e alemã, Ernesto Roma foi o primeiro a “descobrir a América” (Lisboa, 1976) e a importar para Portugal as novidades pioneiras ao nível do tratamento e assistência aos diabéticos.

Entre 1926 e 1946, a ação da APDP teve vários resultados práticos: a abertura de uma consulta da diabetes, o estabelecimento de um programa educativo multifacetado para os doentes, a criação de meios complementares de apoio aos diabéticos e a formação de profissionais de saúde. Esses quatro pontos permitiram estruturar a diabetologia em Portugal no período referido e serviram de base a melhoramentos posteriores. Recorde-se que, nesse período, não existiam consultas especializadas em diabetes fora da APDP.

Somente após a criação da especialidade de endocrinologia-nutrição pela Ordem dos Médicos em 1956 (Vieira, 2018, p.41) foi possível formar médicos especialistas e criar consultas de endocrinologia com profissionais treinados nessa área. Até esse momento milhares de diabéticos eram seguidos por clínicos gerais ou internistas. Por isso, durante anos, só uma minoria tinha acesso a uma assistência especializada em consultas de endocrinologia nos principais hospitais portugueses de Lisboa, Coimbra e Porto (Lisboa, 1976, p.28).

O primeiro ponto a destacar foi a criação de uma consulta específica da diabetes. A maioria dos diabéticos portugueses quando procurava um médico num hospital público ou numa clínica privada era atendida por clínicos gerais ou por médicos especialistas em medicina interna. A primeira consulta da diabetes existente em Portugal foi a da APDP, inaugurada em novembro de 1928 na sede da associação. A consulta era dirigida pelo diretor técnico da APDP, o doutor Ernesto Roma, e contou no início com o auxílio dos médicos António Moraes David e Júlio Lopes (APDP, 1927-1929, p.40). Para se perceber o pioneirismo dessa consulta bastará dizer que a primeira consulta de endocrinologia num hospital português foi criada em novembro de 1946 no Hospital Curry Cabral em Lisboa (Vieira, 2018, p.75-76). Isso significa que a consulta da diabetes da APDP precedeu em quase duas décadas a primeira consulta de endocrinologia num hospital.

As consultas realizavam-se duas vezes por semana, às terças-feiras e aos sábados às 10h da manhã, sendo as vagas preenchidas com base num sistema de senhas limitadas. Porém, para serviço clínico urgente os doentes podiam ser atendidos nos dias úteis das 9h às 19h e inclusive de noite, aos domingos e feriados. Os doentes tinham à disposição um número de telefone que reencaminhava o telefone para a residência do doutor Ernesto Roma. Este ouvia o doente e decidia, se fosse necessário, a assistência ou hospitalização dos doentes pobres (APDP, 1931a, p.5). As consultas da diabetes podiam ser frequentadas por doentes não pobres, mediante o pagamento da respetiva consulta, no valor de cinco escudos, que revertiam a favor da APDP. A consulta da diabetes era complementada com palestras educativas aos doentes. A falta de documentação e de testemunhos neste âmbito impede-nos de saber quais eram os procedimentos da consulta e da dinâmica da prática clínica.

Quando era necessária a hospitalização de algum doente dado o seu estado de saúde grave, a APDP assegurava o internamento dos doentes que acompanhava. A associação estabeleceu um protocolo com a Ordem Terceira de S. Francisco (uma instituição assistencial portuguesa) para garantir o internamento dos diabéticos pobres e indigentes fora da região de Lisboa, colaborando neste processo as câmaras municipais que pagavam parte do transporte e internamento (APDP, 1931a, p.7). Em 1937, o doutor Ernesto Roma solicitou à Comissão Administrativa da APDP autorização para abrir uma consulta especial do pé diabético. O objetivo dessa consulta era observar e tratar os pés e membros inferiores dos doentes, para evitar amputações sépticas e circulatórias devido a gangrenas (APDP, 1929-1938, p.53). Para perceber o vanguardismo dessa consulta do pé diabético criada na APDP em 1937 bastará referir que no Hospital Geral de Santo António, um dos maiores hospitais do país, somente em 2000 apareceu essa consulta de forma diferenciada (Silva, 2003, p.11).

O segundo ponto importante da diabetologia da primeira metade do século XX foi o plano de educação dos diabéticos. O primeiro médico a propor a educação dos diabéticos como parte do seu tratamento foi o americano Elliott Joslin, que Ernesto Roma teve oportunidade de conhecer em Boston e de seguir o seu trabalho.

Joslin deu uma atenção particular à educação dos diabéticos desde a fundação da Joslin Clinic, em 1898. Essa foi a primeira clínica especializada no atendimento de diabéticos no mundo e pôs em prática um programa educativo com o triplo objetivo de educar, cuidar e investigar (Martin, 1958, p.390-391). Essa instituição tinha como lema “*teaching is cheaper than nursing*” (Barnett, 1998, p.45), defendendo que a promoção da educação dos diabéticos passava pelo ensino de regras de vida, higiene, autoinjeção de insulina, recolha de urina, confeção de refeições, entre outras.

Com o mesmo ideal, Ernesto Roma pôs em prática na APDP os mesmos princípios educativos. Antes da consulta médica ter lugar, os doentes assistiam a uma formação sobre dietética e educação médica, assuntos julgados indispensáveis para os doentes compreenderem a sua doença e a dieta a cumprir. No âmbito da educação médica eram abordados temas como: pesquisa da glicose na urina, conhecimento dos elementos que compõem os alimentos (hidratos de carbono, proteínas e gorduras), informação sobre as calorias, conhecimento sobre o órgão em que se localizava a doença e como evitá-la, saber quais os sintomas e causas do coma diabético, saber utilizar uma tabela de alimentos, saber o que era a insulina e como injetá-la, saber quais eram os primeiros sinais da hipoglicémia, entre outros. Ao nível da dietética a formação incluía conhecer os princípios gerais da alimentação e saber como preparar sopas, condutos e acompanhamento, saladas, pão, sobremesas, doces e bebidas (APDP, 1927-1929, p.5). Essa educação dos diabéticos era feita em lições dadas a grupos pequenos em sistema de perguntas-respostas, devendo ser o ensino muito objetivo com a exemplificação de tudo quanto era abordado (APDP, 1931b, p.5).

Em 1931, foi lançado um pequeno livro intitulado *Conselhos aos diabéticos* (Roma, 1931), que resumia a informação das palestras e formações que antecediam as consultas médicas. No mesmo ano, iniciou-se a publicação dos primeiros *Boletins da APDP*, com o objetivo de ser uma publicação periódica específica sobre a diabetes. A sua periodicidade foi, contudo, muito irregular ao longo dos anos e dependente dos condicionalismos financeiros da própria associação. Apenas são conhecidos sete números publicados para o período aqui estudado (Vieira, 2020, p.154), mas temos que assinalar que se tratou de uma publicação muito útil na divulgação dos princípios de educação aplicados à diabetes.

Quer as consultas quer as ações educativas para os diabéticos eram complementadas com apoios variados, como o laboratório de exames, a farmácia e a cozinha dietética. O laboratório de análises clínicas começou a funcionar em 1929, quando a Comissão Administrativa chegou a acordo com dois médicos analistas do Laboratório de Clínica Médica da Faculdade de Medicina da Universidade de Lisboa para a montagem e exploração de um laboratório clínico (APDP, 1927-1929, p.44v). Sob direção do doutor Moraes David e do doutor Carlos Trincão eram realizados exames clínicos à urina e sangue ao que se juntaram exames bromatológicos à composição química dos alimentos, estes últimos sob direção do farmacêutico militar José Pedro Alves, que à data era chefe do Laboratório da Manutenção Militar. Essas colaborações com médicos do laboratório da Faculdade de Medicina e com um farmacêutico do laboratório da Manutenção Militar mostram o cuidado da APDP em estabelecer parcerias de qualidade, que tinham por trás a chancela de reputadas instituições académica e militar. Apesar de a APDP ter tido ao longo das primeiras décadas de existência várias limitações financeiras, tema que não exploraremos neste estudo, não foi impeditivo de manter parcerias que beneficiavam diretamente os doentes seguidos por essa associação.

Mais tarde o laboratório juntou outras valências: reações serológicas e exames bacteriológicos e parasitológicos, análise de líquido cefalorraquidiano, pus, exsudados, secreções pulmonares, suco gástrico e fezes. Desse modo alargou o seu âmbito de atuação e estava aberto tanto aos sócios da APDP como à comunidade, encontrando na prestação desses serviços um meio complementar de angariação de fundos para sobreviver.

É importante assinalar ainda a criação de uma cozinha dietética. Como já dissemos, as palestras aos diabéticos realizadas por Ernesto Roma incluíam informação sobre dietética, mas houve necessidade de tornar mais prática a formação. A procura crescente levou Ernesto Roma a solicitar à Comissão Administrativa a instalação de fogões para ensinar culinária dietética (APDP, 1929-1938, p.56v).

A estruturação da diabetologia fez-se também à custa da formação de profissionais, em particular dos profissionais ligados à saúde. O aparecimento dos primeiros estudos endocrinológicos a partir da década de 1930 e 1940 começou a produzir um conjunto de conhecimentos mais sólidos acerca da diabetes. Deve assinalar-se a publicação das primeiras revistas sobre endocrinologia, publicação conjunta dos endocrinologistas portugueses e espanhóis. Foi o caso da *Acta Endocrinológica Ibérica* e da *Revista Luso-espanhola de Endocrinologia e Nutrição*. Essas revistas permitiram a divulgação de conhecimentos relativos à diabetes. Mas no que dizia respeito ao nível assistencial, a APDP manteve-se sempre na frente, incluindo esses conhecimentos na formação de profissionais de saúde.

A ideia de formar e qualificar profissionais envolvidos de alguma forma no tratamento ou assistência aos diabéticos já existia no estrangeiro. Desde o início da atividade da APDP houve um certo cuidado na formação dos profissionais, embora somente na década de 1930 tenha sido possível começar a concretizar esse desiderato.

Em 1935, a APDP decidiu iniciar um programa de visitas ao domicílio, recorrendo a empregadas treinadas para o efeito. Essas funcionárias iam à casa dos doentes avaliar o seu estado de saúde e a forma como estava a ser observado o tratamento, entre outros aspetos. De destacar que as visitas serviriam para verificar se o mesmo diabético teria pessoas com o “dever moral” de o auxiliar, fossem família ou particulares (APDP, 1929-1938, p.70).

Os anos de 1937 e 1938 foram marcados por diversas iniciativas no plano da formação de profissionais. Em setembro de 1937, uma colaboração entre a APDP e a Cruz Vermelha permitiu uma formação sobre a técnica do maqueiro, o transporte de doentes e feridos e as gazes de guerra. No final do ano o diretor clínico solicitou autorização para abrir consulta de quiropodia para tratamento do pé diabético a cargo de uma profissional de nacionalidade alemã de nome Hilde, que passou a dar também formação a outros profissionais de saúde (APDP, 1929-1938, p.50, 53).

Em 1938, o diretor clínico decidiu juntar um serviço de histologia e anatomia patológica às valências de que a APDP já dispunha. Para tal, custeou a formação de uma médica, a doutora Colette Macedo Marques, tendo esta frequentado o Curso de Histologia e Anatomia Patológica com a finalidade de garantir esse serviço, e solicitou em 1939 ao Ministério da Guerra autorização para a mesma frequentar o Laboratório de Análises da Manutenção Militar por forma a proporcionar-lhe formação na realização de exames bromatológicos. Ernesto Roma propôs também o início das visitas pelas empregadas da APDP em vários distritos de Portugal como nas cidades de Viseu e de Bragança, duas cidades do interior do país.

Em 1940, Ernesto Roma organizou uma série de conferências médicas com vista a estimular o estudo da diabetes e impulsionar essa área, da mesma forma que contratou uma enfermeira norte-americana, de nome Sarah Norton, para dar formação especializada em enfermagem (Veloso, Correia, 2017, p.252).

Embora Portugal não tivesse participado na Segunda Guerra Mundial, as restrições de guerra dificultaram os trabalhos da APDP. Houve mesmo um período em que foi necessário racionar a insulina, fazer o aproveitamento de todas as sobras dessa substância e conseguir esse produto a todo o custo. Habilmente, Ernesto Roma soube tirar partido da sua influência junto da Cruz Vermelha Portuguesa, da qual era membro, exercendo influência para adquirir insulina via Cruz Vermelha Americana, tendo recebido 25 mil tubos de insulina, 1.200 agulhas e 500 seringas (CVP, 1946, p.87) num período particularmente difícil em termos de abastecimento e circulação de produtos em termos internacionais. O recurso à Cruz Vermelha Americana já havia sido feito por Portugal para a obtenção de penicilina (Bell, Pita, Pereira, 2017).

A partir de 1947, a situação financeira da APDP tornou-se muito grave. A falta de recursos levou a APDP a um quase encerramento, tendo sido nomeada uma Comissão Liquidatária. A intervenção de Joaquim Dinis da Fonseca, que tinha sido subsecretário de Estado da Assistência Social e das Finanças e deputado à Assembleia Nacional (Veloso, Correia, 2017, p.252), permitiu melhorar a situação, superar as dificuldades e iniciar uma nova fase na vida da associação.

## Considerações finais

A APDP foi a principal instituição dedicada à diabetes existente em Portugal até meados do século XX. Revelou-se um projeto médico-social pioneiro em Portugal e, tanto quanto sabemos, no mundo. Na década de 1920 a diabetes estava longe de ser considerada um problema de saúde pública, mas a APDP considerava-o como tal e empreendeu esforços para socorrer os diabéticos sem meios para assegurarem o seu tratamento.

Na primeira fase da existência da associação, entre 1926 e 1946, a organização da saúde e assistência em Portugal era supletiva e globalmente limitada. A posição do Estado português em relação à assistência e aos cuidados de saúde determinou que uma parte da sociedade portuguesa dependesse da caridade e da beneficência prestada por instituições particulares. Após a Segunda Guerra Mundial, enquanto vários países europeus começaram a organizar o seu Estado-providência, em Portugal o acesso aos cuidados de saúde pela população dependeu da ação dos poderes locais e das misericórdias, que geriam boa parte dos hospitais e instituições de solidariedade social. O acesso aos cuidados prestados em clínica privada só estavam ao alcance dos estratos socioeconómicos mais favorecidos. Contudo, mesmo os trabalhadores com acesso à designada Previdência Social do Estado Novo não encontravam nas estruturas estatais assistência especializada e diferenciada no campo da diabetes.

A APDP foi por isso, durante duas décadas, o principal polo dedicado ao estudo, tratamento, vigilância, assistência, educação e formação de profissionais ligados à diabetes. Mesmo com o aparecimento do Serviço Nacional de Saúde em Portugal, após a revolução de 25 de abril de 1974, a APDP foi chamada a colaborar com o seu conhecimento e capital técnico-científico. Prestou apoio e colaboração técnica aos Serviços de Saúde Pública na educação sanitária da população, no rastreio e diagnóstico precoce da doença e na organização de cursos para médicos e enfermeiros.

Podemos afirmar com toda a certeza que a construção e o desenvolvimento da diabetologia em Portugal na primeira metade do século XX se deveu à Associação Protetora dos Diabéticos Pobres. A APDP foi influenciada pelo modelo americano de luta contra a diabetes e colocou em prática um programa inovador de combate a essa doença, alicerçada na educação dos diabéticos. Atendendo a que os doentes assistidos pela APDP tinham poucos recursos económicos, o foco do processo educativo passava por conferir autonomia aos doentes, ensinando a autoanalisar a urina, a dominar a técnica de autoinjecção de insulina, a planear a dieta, a quantificar e a confecionar os alimentos. A educação dos diabéticos permitiu certo empoderamento dos doentes, que prescindiam de cuidados médicos diários e continuados, ao mesmo tempo que permitia uma reintegração social dos diabéticos.

A luta contra a diabetes no período considerado esbarrou com um crónico subfinanciamento por parte das entidades estatais. Essa situação atrasou várias iniciativas médico-assistenciais e manteve um déficit assistencial antidiabético. O país não estava uniformemente coberto por uma rede de assistência aos diabéticos. Para além de Lisboa, a existência de filiais de luta contra esse problema dependia da disponibilidade e da organização dos diferentes locais. O financiamento disponível era reduzido e tinha por base as quotas de sócios, as doações de cidadãos, os protocolos com entidades e algumas iniciativas como a organização de festas para angariar dinheiro.

Como evidenciamos, a estruturação da diabetologia portuguesa teve como ponto de partida o plano traçado pela APDP e pelo seu mentor Ernesto Roma com o estabelecimento de consultas, o apoio material aos diabéticos, a educação e a formação de profissionais de saúde capazes de intervir de forma diferenciada junto dos doentes. Apesar de a documentação existente não permitir perceber com clareza em que consistiu o programa de formação de profissionais de saúde, bem como outros aspetos, foi esse plano que permitiu, a partir da década de 1950, formar alguns médicos que prestavam serviço na APDP e que depois transportaram os seus conhecimentos para os respetivos serviços hospitalares e, desse modo, ampliaram a oferta especializada.

Não tivemos a pretensão de destacar Ernesto Roma de um ponto de vista que alguns possam considerar hagiográfico. O papel vanguardista desse médico na estruturação de um plano de impulsionamento da diabetologia em Portugal é indiscutível. Os fatos históricos são prova disso. Falamos do estabelecimento da primeira associação de proteção e tratamento aos diabéticos do mundo, da criação de uma consulta da diabetes e de outros mecanismos assistenciais que antecederam em décadas as primeiras consultas da diabetes nos principais hospitais portugueses, na constituição de um capital técnico e científico que ajudou a formar pessoal médico, de enfermagem, de nutrição etc., ao longo de décadas, salientando-se a ajuda que deu no plano da luta contra a diabetes aquando da criação do Sistema Nacional de Saúde em Portugal a partir de 1974. Nesse sentido não há como fugir ao incontornável contributo de Ernesto Roma, em especial para o período aqui estudado.

Neste estudo, reportamo-nos essencialmente ao contributo dessa associação até 1946. A instituição continua a ser uma referência em Portugal no campo da diabetologia, nas mais diversas valências, mantendo uma presença forte junto da sociedade portuguesa e perseverando no legado do seu mentor, hoje também por meio da Fundação Ernesto Roma.^3^

## Data Availability

Não estão em repositório.

## NOTAS

1 Estado Novo é a designação dada ao regime ditatorial que vigorou em Portugal resultante da revolução de 28 de maio de 1926 até a revolução de 25 de abril de 1974.

2 O acesso ao Arquivo Histórico dos Hospitais Civis de Lisboa está atualmente interdito pelas débeis condições de segurança das infraestruturas, o que não permitiu a consulta da documentação relativa ao Hospital de Santa Marta.

3 Informações mais detalhadas acerca da associação podem ser consultadas no *website* da APDP, disponível em https://apdp.pt/, e da Fundação Ernesto Roma, disponível em https://www.fundacaoernestoroma.org/.
